# Supplementary material for: Exposure to authoritarian values leads to lower positive affect, higher negative affect, and higher meaning in life
Source: PLoS One. 2021 Sep 15;16(9):e0256759. doi: 10.1371/journal.pone.0256759 (PMC8443031; doi:10.1371/journal.pone.0256759)
Supplement: S1 File — (DOCX) [file pone.0256759.s001.docx]

**Supplemental Materials**

**The Affective and Existential Effects of Hitler: Exposure to Authoritarian Values Leads to Lower Positive Affect, Higher Negative Affect, and Higher Meaning in Life**

**Contents:**

**Pilot Testing Original Passages**, Preliminary Study and Studies 2-5, page 2

**Preliminary Study Methods and Results**, pages 4-12

**Study 2 Pilot Study A**, pages 13-17

**Study 2 Pilot Study B**, pages 18-21

**Supplemental Analyses**, Study 2, pages 22-31

**Full Text of All Manipulation Materials**, Study 2, pages 32-33

**Overview of Supplemental Analyses**, Studies 3-5, page 34

**Study 3 Supplemental Analyses**, pages 35-37

**Study 4 Supplemental Analyses**, pages 38-40

**Study 5 Supplemental Analyses**, pages 41-46

**Condition Effects on Mood**, Studies 3-5, page 47

**Moderation by Conservatism**, Studies 3-5, pages 48-50

**Testing Moderation of Condition Effects on Meaning by Mood and Covariates,** Preliminary Study and Studies 3-5, pages 51-55

**Testing Moderation of Condition Effects on Message Evaluations,** Preliminary Study and Studies 3-5, pages 56-57

**Results for Moral Superiority**, Studies 2-5, pages 58-60

**Condition Effects on Mood and Meaning in Life**, Aggregated Data for Studies 3-5, page 61

**Further Probing Anger and (dis)Agreement**, Aggregated Data for Studies 3-5, pages 62-65

**Pilot Testing for Original Passages Preliminary Study and Studies 2-5 Stimulus Materials.**

MIL has been shown to be affected by processing fluency (Trent, Lavelock, & King, 2013). As such, we tested whether the authoritarian passage we crafted might be more easily processed. We analyzed the original passages for ease-of-reading via Microsoft Word. The Flesch-Kincaid grade level statistics suggested that all passages were of a similar reading level, although the authoritarian passage (grade level = 14.3) was at a slightly higher reading level than the egalitarian (grade level = 12.2) and control (grade level = 10.5) passages. As such, processing fluency differences across conditions were unlikely to provide an explanation for the predicted effects.

Additionally, we conducted an initial pilot test of the passages (*N*=74). Participants were randomly assigned to read the authoritarian, egalitarian, or control passage, and then evaluated the passage on a number of dimensions. These included, “How hostile was the author of this passage; How certain was the author of this passage; How likely is it that the author of this passage would change their mind; How much will this author’s philosophy of life help them make decisions; How much will this author’s philosophy of life help them make the right choices; Did the author of this passage sound like a leader; If you were interacting with the author of this passage; would it be easy to predict their behavior; Did the author of this passage sound like an authority?”

We submitted each item to an ANOVA, and found that the authoritarian author was rated as more hostile, less warm, less of a leader, less apt to make the right choice, less trustworthy, and that the authoritarian passage made less senses, *F*’s(2,71) ranged from 3.36 to 10.39, *p*’s ranged from .04 to .001, partial η^2’s^ ranged from .07 to .93. None of these items was associated with MIL. Next, we submitted MIL to an ANCOVA, controlling for each of these items, and they did not wipe out the effects of condition on MIL, *F*(2,63) = 3.72, *p* = .03, partial η^2^ = .11. Although this was a small pilot study, as in Studies 2-5, the authoritarian condition lead to higher MIL, *M*(*SD*) = 5.78(0.98), than the control, *M*(*SD*) = 4.75(1.57), and egalitarian, *M*(*SD*) = 4.38(1.77), conditions.

**Preliminary Study**

**Methods**

**Participants and Procedure**.

1611 participants in the United States were recruited on MTurk to participate in an online study for $0.15. Participants were 64% women, 74.3% White/European American, 9.7% Black/African American, 6.8% Asian, 6.3% Latino(a), 0.7% Native American, and 2.2% selected “other.” Ages ranged from 18-98, *M*(*SD*)=35.30(12.18). Incomes ranged from $15,000 to over $151,000, and median income was $50,001-$75,000. Modal education was “some college” and 90.7% of participants had completed some college or more. The measures, materials, and procedure were identical to Studies 3-5, except we counterbalanced MIL with 3-items measuring RWA.

**Results**

Correlations among all measures are shown in Table S1. Results for mood, evaluations, and moral superiority are shown in Table S2. The egalitarian condition led to higher positive mood than the control condition, which was higher than the authoritarian condition. The authoritarian condition was significantly higher on NA than the egalitarian and control conditions. The authoritarian passage led to poorer evaluations and greater moral superiority. As shown in Table S3, in support of our prediction, exposure to authoritarian values led to higher meaning than egalitarian and control perspectives, controlling for mood. Controlling for all covariates, condition effects on meaning in life remained significant: Study 2, *F*(2,1596)=5.00, *p*=.007, *d*=0.20;

**Condition effects on RWA.** Participants in this Preliminary Study completed 3 RWA items (counterbalanced with MIL) following the manipulation. The effect of condition on RWA was significant, *F*(2, 1609) = 4.33, *p* = .013, partial η^2^ = .07. In the absence of consideration of covariates, the condition means suggested that only the egalitarian condition, *M*(*SD*) = 3.41(1.57), differed from the other two; for control, *M*(*SD*) = 3.65(1.53), for authoritarian, *M*(*SD*) = 3.66(1.52). We submitted RWA to an ANCOVA controlling for PA, NA, target evaluations, moral superiority, and religiosity. Controlling for PA [*F*(1,1525) = 0.01, *p* = .95, partial η^2^ = .000] and NA [*F*(1, 1525) = 11.11, *p <* .001, partial η^2^ = .012], target evaluations, [*F*(1, 1525) = 3.12, *p =* .08, partial η^2^ = .006], moral superiority, [*F*(1, 1525) = 7.61, *p =* .006, partial η^2^ = .004], and religiosity, [*F*(1, 1525) = 373.19 *p <* .001, partial η^2^ = .20], the effect of condition remained significant, *F*(2, 1525) = 11.31, *p <* .001, partial η^2^ = .07. The corrected means are shown in Figure S2. As can be seen, scores on the short RWA measures were higher in the authoritarian condition than in the other two groups.

The pattern of results for RWA and MIL were quite similar. Do fluctuations in RWA explain the condition effect on MIL? They do not. When MIL was regressed on the covariates, RWA, condition dummies, and the interactions between RWA and conditions, the main effects for the authoritarian condition dummy, β = .09, *p* = .006, and RWA, β=.15, *p*<.001 remained significant.

When RWA was regressed on the covariates and MIL, the egalitarian dummy remained related to RWA, for the egalitarian dummy, β = -.27, *p* = .002; and the authoritarian dummy was marginally significant, β = .16, *p* = .09. MIL did not interact with condition dummies to predict RWA, both *p*’s > .24. Thus, although both RWA and MIL were affected by conditions in a similar way, the effects of condition on these variables were independent of each other.

**Conservatism.** We tested for moderation by conservatism. For the main effects of the authoritarian condition, β = .10, *p* = .002, egalitarian condition, β = -.02, *p* = .52, and conservatism, β = .14, *p* < .001. For the conservatism X authoritarian condition interaction, β = -.07, *p* = .049. (The conservatism X egalitarian condition interaction was non-significant, β = .02, *p* = .55). Note again, that the negative sign of the conservatism X authoritarian dummy does not indicate that the manipulation was stronger for those high in conservatism. A graph of the generated regression lines, predicting MIL for those high and low on conservatism is shown in Figure S3. As can be seen, condition effects were strongest among those who were low in conservatism.

**Demographics**. We conducted exploratory tests for moderation by demographics. A 2 (gender, women or men) X 3 (condition) ANCOVA controlling for PA, *F*(1,1512) = 47.74, η^2^=.03, and NA *F*(1,1512)=23.77, η^2^=.02, both *p*’s <.001 showed that a significant main effect of condition, *F*(2,1512)=8.29, η^2^=.001, *p*<.001. For the Gender X Condition interaction, *F*(2,1514)=3.60, η^2^=.011, *p*=.027. Examination of the means showed that although both men and women reported highest levels of MIL in the authoritarian condition, among women, the egalitarian condition led to lower MIL than in the control condition. However, this effect did not replicate across other the studies.

Subsequent analyses testing for moderation by demographics controlled for the effects of mood. Neither ethnicity nor education moderated the effects of condition on MIL, both *p*’s > .17, although there was a significant main effect of education, *F*(1,1525) = 9.59, *p* =.002, η^2^=.005. Income and age were treated as continuous variables, mean centered, and used to compute interaction terms with the condition dummies. Neither income nor age demonstrated evidence of moderation (all *p*’s > .28), but there were significant main effects for each, both β’s = .16, both *p’s <* .001. Religiosity failed to show evidence of moderation, *p*’s > .09.

***Table S1. Correlations Among Measures, Preliminary Study***

|  | Positive Affect | Negative Affect | Message Evaluation | Moral Superiority | RWA | Intrinsic Religiosity |
| --- | --- | --- | --- | --- | --- | --- |
| MIL | .16** | -.10** | .06 | .08* | .18** | .30** |
| PA |  | -.08* | .53** | .04 | .10** | .14** |
| NA |  |  | -.38** | .34** | .11** | -.01 |
| ME |  |  |  | -.27** | .11** | .11** |
| MS |  |  |  |  | .07 | .08 |
| RWA |  |  |  | . |  | .45** |

***Note***. *N*=1609; **p*<.004; ***p*<.001. PA= positive affect; NA= negative affect; ME= message evaluations; MS= moral superiority; RWA = rightwing authoritarianism.

***Table S2. Effects of Condition on Mood and Message Evaluations, Preliminary Study***

|  | **Control** | **Egalitarian** | **Authoritarian** | **Effect of Condition** |
| --- | --- | --- | --- | --- |
| *n*’s | 535 | 544 | 532 |  |
| PA | 3.18(1.54)_a_ | 3.64(1.53)_b_ | 2.67(1.57)_c_ | *F*(2,1600)=52.99,** *d*=0.51 |
| NA | 1.76(1.23)_a_ | 1.86(1.14)_a_ | 2.66(1.60)_b_ | *F*(2,1600)=93.85,** *d*=0.70 |
| ME | 5.01(1.15)_a_ | 5.24(1.46)_b_ | 3.86(1.24)_c_ | *F*(2,1600)=176.46,** *d=*0.94 |
| MS | 2.64(1.57)_a_ | 2.53(1.51)_a_ | 3.12(1.66)_b_ | *F*(2,1600), 21.28,** *d*=0.35 |

***Note***. PA= positive affect; NA=negative affect; ME=message evaluation; MS=moral superiority

***Table S3. Effects of Condition on Meaning in Life, Preliminary Study***

|  |  | **Control** | **Egalitarian** | **Authoritarian** | **Effect of Condition**  **& Effect of Condition controlling for mood** |
| --- | --- | --- | --- | --- | --- |
|  | *n*’s | 535 | 544 | 532 |  |
| Raw *M*(*SD*) |  | 4.72(1.31) | 4.70(1.34) | 4.79(1.32) | *F*(2, 1608)=0.62 |
| *M* adjusted for mood |  | 4.68_a_ | 4.61_a_ | 4.93_b_ | *F*(2, 1598)=7.49*,***  *d*=0.20 |

***Figure S1. Distribution Density Plot for Meaning in Life by Condition, Preliminary Study***


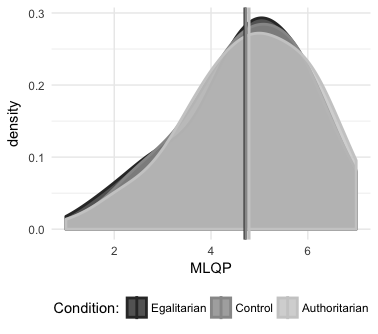


***Figure S2.* *Post-manipulation RWA as a Function of Condition, Preliminary Study. Error bars are 95% CIs***

***Figure S3. Meaning in Life as a Function of Condition and Conservatism, Preliminary Study***

**Study 2 Pilot Study A**

**Overview**

In order to select final stimulus materials for the manipulation in Study 2, we first brainstormed historical authoritarian and egalitarian leaders and locating transcripts of speeches from them. Once we identified speeches, we selected excerpts that most parsimoniously expressed authoritarian or egalitarian values. The purpose of Pilot Study A was to ensure that these speeches were similar on dimensions that might influence meaning (mood and familiarity), and to verify that authoritarian speeches actually represented authoritarian values.

**Method**

**Participants and Procedure**

We recruited 518 participants from Amazon Mechanical Turk. Age, *M(SD*)=38.54(12.24) ranged from 19 to 80. Participants were 60.8% female, 77.2% White/European American, 9.7% Black/African American, 5.7 Asian, 5.0% Hispanic/Latinx, and 2.4% indicated “other.” Median income was $35,001-$50,000. Modal education was a Bachelor’s Degree.

Participants were first instructed,

“We are interested in the ways people perceive and remember information about another person. You will be seeing a response to a question given by someone in a previous study. This person was asked to complete a brief writing task, based on an image prompt, and we are going to show you the response that was given. The image and response are from a very old study of the Thematic Apperception Test, so the language may seem antiquated and unusual.

While reading, try to suspend judgment and just learn about this person’s experience. Try to focus on the ideas conveyed without immediately judging the author.  Please pay close attention because later we will be testing your memory of the information communicated.”

Then, they were briefly shown a Thematic Apperception Test image of a ship captain before being randomly assigned to view what was ostensibly the former participant’s response to the image. These responses consisted of excepts selected from the speeches of real-world authoritarian (Adolf Hitler (*n*=53), Vince Lombardi (*n*=51), George Patton (*n*=52), Benito Mussolini (*n*=53), Kim Jong Un (*n*=53), and Josef Stalin, (*n*=52)), and egalitarian leaders (Bernie Sanders (*n*=50), Albert Einstein (*n*=51), Greg Popovich (*n*=51), Martin Luther King Jr. (*n*=52)). In some cases, the original text of these speeches was slightly altered in order to make them more consistent with a conceptual response to the image, as well as to reduce the antiquity of the language. Full excerpts used in the study can be found in Appendix A. Links to the original transcripts of each speech can be found in Appendix C. In no case did our alterations change the meaning of the speaker’s message, or content conveyed by the speech.

**Measures**

All items were rated on a scale from 1 (*not at all/strongly disagree*) to 7 (*very much/strongly agree*). Immediately after reading one of these speeches, we measured positive and negative affect by asking participants to rate face-valid mood descriptors: For positive mood, *M(SD*)=2.91(1.89), *α=.*93, and negative mood, *M(SD*)=2.54(1.56), *α=.*92*.* Next, in order to confirm that the authoritarian speeches represented authoritarian values, and that egalitarian ones did not, participants were instructed to rate 6 items measuring RWA used in Study 2, *M(SD)=*4.63(1.50)*, α=*.90*,* and 4 items from the Social Dominance Orientation Scale (Ho et al., 2015), *M(SD)=*4.02(1.73)*, α=*.88*,* as they thought author of the passage would rate them. Lastly, participants rated the familiarity of the passage, *M(SD)=*2.65(1.84).

**Results**

Table S4 shows cell means for each variable. As expected, authoritarian speeches generally led to lower positive affect and higher negative affect than egalitarian speeches. The Lombardi speech was anomalous among the authoritarian speakers, leading to relatively high positive mood. Among egalitarian speakers, Martin Luther King Jr. engendered relatively high positive affect, and he and Einstein led to low negative affect, relative to other egalitarian authors. Confirming our assumption that the speeches of authoritarian leaders represented authoritarian values, and those from egalitarian speakers did not, all authoritarian speeches led participants to indicate they thought the author would endorse right-wing authoritarianism and social dominance orientation more strongly than egalitarian authors. Among the authoritarian speakers, the means for Patton were especially high. Lastly, most speeches were similar on familiarity. Sanders, the most contemporary speaker who Americans presumably have more exposure to, was rated a more familiar than the rest of the speakers. Hitler, Patton, and Kim Jong Un were rated as the least familiar.

**Discussion**

The results of this pilot confirmed that real-world authoritarian speeches convey authoritarian values, and real-world egalitarian speeches do not. Exposure to real-world statements of authoritarian values engendered worse mood than egalitarian speeches. Based on the results of this pilot, we determined to retain the speeches of Hitler, Patton, and Kim because they were the least familiar. Additionally, we retained Stalin’s speech in the second pilot because this condition conformed to a similar pattern, and he is conceptually emblematic of authoritarian leadership. Relying on these passages would allow us to avoid the potentially confounding effects of familiarity on meaning in life. However, excluding Einstein, each egalitarian speech was unusual. Sanders was especially familiar, Popovich engendered atypically high negative affect, and Martin Luther King Jr. led to especially strong effects on mood. Thus, we conducted a second pilot study to test another batch of potentially less familiar egalitarian speeches.

***Table S4.*** ***Means for Each Speech on Each Variable, Pilot A***

|  | Hitler  *M(SD*), CI | Patton  *M(SD*), CI | Un  *M(SD*), CI | Stalin  *M(SD*), CI | Mussolini  *M(SD*), CI | Lombardi  *M(SD*), CI | Sanders  *M(SD*), CI | Einstein  *M(SD*), CI | Popovich  *M(SD*), CI | King Jr.  *M(SD*), CI |
| --- | --- | --- | --- | --- | --- | --- | --- | --- | --- | --- |
| PA | 2.24(1.62)  [1.78, 2.70] | 2.23(1.72)  [2.74, 2.71] | 2.83(1.83)  [2.33, 3.34] | 2.76(1.89)  [2.23, 3.29] | 2.46(1.89)  [1.93, 2.99] | 3.36(1.82)  [2.85, 3.88] | 3.37(1.81)  [2.85, 3.88] | 3.37(2.06)  [2.75, 3.98] | 2.86(1.79)  [2.35, 3.37] | 3.72(1.72)  [3.23, 4.20] |
| NA | 2.73(1.74)  [2.24, 3.23] | 3.29(1.54)  [2.85, 3.72] | 2.60(1.49)  [2.19, 2.01] | 2.82(1.59)  [2.38, 3.27] | 2.91(1.54)  [2.48, 3.34] | 2.37(1.56)  [1.93, 2.81] | 2.37(1.56)  [1.92, 2.81] | 1.63(0.96)  [1.34, 1.91] | 2.79(1.55)  [2.35, 3.23] | 1.82(1.27)  [1.46, 2.18] |
| RWA | 5.28(1.36)  [4.90, 5.66] | 5.79(1.02)  [5.50, 6.08] | 4.94(1.60)  [4.50, 5.38] | 5.05(1.26)  [4.70, 5.40] | 5.10(1.39)  [4.71, 5.49] | 5.07(1.04)  [4.78, 5.36] | 3.64(1.19)  [3.30, 3.98] | 3.78(1.57)  [3.32, 4.25] | 3.97(1.39)  [3.57, 4.37] | 3.61(1.14)  [3.29, 3.93] |
| SDO | 4.80(1.75)  [4.30, 5.30] | 5.48(1.43)  [5.08, 5.88] | 4.34(1.70)  [3.88, 4.81] | 4.64(1.40)  [4.25, 5.03] | 4.50(1.70)  [4.02, 4.98] | 4.48(1.45)  [4.07, 4.89] | 2.98(1.12)  [2.66, 3.29] | 2.73(1.43)  [2.31, 3.16] | 3.15(1.42)  [2.74, 3.55] | 2.89(1.29)  [2.53, 3.25] |
| Familiarity | 2.08(1.46)  [1.67, 2.49] | 2.51(1.82)  [2.00, 3.02] | 2.36(1.77)  [1.87, 2.85] | 2.62(1.84)  [2.10, 3.13] | 2.86(1.98)  [2.31, 3.42] | 2.92(1.96)  [2.37, 3.47] | 3.26(2.08)  [2.67, 3.85] | 2.59(1.75)  [2.07, 3.11] | 2.62(1.82)  [2.10, 3.14] | 2.71(1.80)  [2.20, 3.21] |

***Note***. PA=positive affect; NA=negative affect; RWA=right-wing authoritarianism; SDO=social dominance orientation; SA=self-authored

**Study 2 Pilot Study B**

**Overview**

There were several purposes to our second pilot study. First, we compared the speeches of authoritarian leaders to a different set of egalitarian speeches. Second, we examined the similarity of the passages we authored to these real-world expressions of authoritarian and egalitarian values. We additionally further probed the extent to which the passages were easy to process to ensure there existed no systematic differences across authoritarian and egalitarian speeches on this dimension.

**Method**

**Participants and Procedure**

Participants were 585 Amazon Mechanical Turk workers. Age, *M(SD*)=37.31(12.42) ranged from 19-80. Participants were 65.8% women, 71.8% White/European American, 12% Black/African American, 7.7% Asian, 5% Hispanic/Latinx, and 3.4% indicated “other.” Median income was $35,001-$50,000. Modal education was a Bachelor’s degree. This study followed the same procedure as Pilot Study A. The only difference was in the speeches that we randomly assigned participants to read. For the authoritarian speeches, participants either read an excerpt from Hitler (*n*=49), Patton (*n*=52), Un (*n*=52), Stalin (*n*=52), or the passage we crafted (*n*=54). For egalitarian speeches, participants either read an excerpt from Eleanor Roosevelt (*n*=47), Mikhail Gorbachev (*n*=48), Dietrich Bonhoeffer (*n*=51; a German anti-Nazi contemporary of Hitler), Einstein (*n*=53), or the passage we authored (*n*=50). We also included a control passage we authored (*n*=49). We included these passages with the goal of selecting three authoritarian and egalitarian speeches (in addition to those we authored) for Study 2.

**Measures**

After reading one of these passages, participants completed the same measures of positive affect, *M(SD*)=3.06(1.81), *α=.*92, and negative affect, *M(SD*)=2.49(1.50), *α=.*91, as in Pilot A. Again instructing participants to rate the items as though they were the author of the speech that they read, participants completed the same measures of right-wing authoritarianism, *M(SD*)=4.38(1.67), *α=.*88, and social dominance orientation, *M(SD*)=3.82(1.67), *α=.*88, as in Pilot A. Finally, participants evaluated the passage on a number of dimensions. These included familiarity, *M(SD)=*2.74(1.76); during what year they thought the passage was written (on a scale from 1930-2018), *M(SD)=*1961(23); and, how easy it was to understand the passage, to read the passage, and how much the content of the passage matched the image, which were aggregated to form an ease of processing composite, *M(SD)=*4.65, *α=.*73.

**Results**

Means for each speech on each variable are shown in Table S5. As expected, authoritarian speeches generally led to lower positive affect and higher negative affect than egalitarian and control statements. Participants again indicated they thought authoritarian speakers would more strongly endorse right-wing authoritarianism and social dominance orientation than egalitarian speakers. Authoritarian and egalitarian speeches were similar on familiarity. Einstein was the most familiar among egalitarian speakers, and Stalin was the most familiar among authoritarians. On average, participants thought all passages were from the 1950s and 60s. Egalitarian passages were rated as more recent than authoritarian passages. Lastly, ease of processing did not appear to differ systematically across authoritarian and egalitarian messages.

**Discussion**

The results of Pilot Study B add confidence to our assertion that authoritarian speakers convey greater authoritarian values than egalitarian speakers. Authoritarian messages led to worse mood than egalitarian perspectives. Egalitarian messages were thought to be more recent, perhaps because people generally believe society has progressed to greater egalitarianism. Authoritarian and egalitarian speeches were also similarly easy to process. Stalin and Einstein were atypically familiar, so these were not retained for the final study materials.

***Table S5. Means for Each Speech on Each Variable, Pilot B***

|  | Hitler  *M(SD*), CI | Patton  *M(SD*), CI | Un  *M(SD*), CI | Stalin  *M(SD*), CI | Auth SA  *M(SD*), CI | Control  *M(SD*), CI | Egal SA  *M(SD*), CI | Roosevelt  *M(SD*), CI | Gorbachev  *M(SD*), CI | Bonhoeffer  *M(SD*), CI | Einstein  *M(SD*), CI |
| --- | --- | --- | --- | --- | --- | --- | --- | --- | --- | --- | --- |
| PA | 2.72(1.93)  [2.23, 3.21] | 2.71(1.68)  [2.23, 3.18] | 2.67(1.72)  [2.19, 3.15] | 2.37(1.59)  [1.90, 2.84] | 2.80(1.77)  [2.33, 3.27] | 3.25(1.66)  [2.76, 3.74] | 3.99(1.72)  [3.51, 4.48] | 3.34(1.75)  [2.84, 3.84] | 3.78(1.80)  [3.28, 4.28] | 2.91(1.89)  [2.43, 3.40] | 3.21(1.86)  [2.73, 3.68] |
| NA | 3.06(1.68)  [2.65, 3.47] | 2.92(1.63)  [2.53, 3.32] | 2.50(1.49)  [2.11, 2.90] | 2.68(1.60)  [2.29, 3.07] | 2.77(1.67)  [2.38, 3.16] | 2.38(1.48)  [1.97, 2.79] | 1.88(1.24)  [1.47, 2.28] | 2.58(1.39)  [2.16, 3.00] | 2.17(1.20)  [1.75, 2.58] | 2.36(1.38)  [1.96, 2.77] | 2.03(1.27)  [1.63, 2.42] |
| RWA | 4.93(1.42)  [4.57, 5.29] | 5.31(1.64)  [4.96, 5.66] | 4.81(1.31)  [4.46, 5.16] | 5.16(1.13)  [4.82, 5.51] | 5.30(1.20)  [4.96, 5.64] | 3.76(1.06)  [3.41, 4.12] | 3.43(1.30)  [3.09, 3.78] | 3.68(1.21)  [3.31, 4.04] | 3.61(1.42)  [3.25, 3.97] | 4.27(1.25)  [3.92, 4.62] | 3.72(1.14)  [3.37, 4.07] |
| SDO | 4.72(1.51)  [4.31, 5.13] | 5.05(1.94)  [4.66, 5.45] | 4.18(1.63)  [3.79, 4.58] | 4.58(1.44)  [4.18, 4.97] | 4.52(1.57)  [4.13, 4.91] | 2.94(1.12)  [2.53, 3.34] | 2.59(1.23)  [2.19, 2.99] | 3.00(1.33)  [2.59, 3.42] | 3.36(1.43)  [2.95, 3.78] | 3.82(1.41)  [3.41, 4.22] | 3.07(1.39)  [2.67, 3.47] |
| Familiarity | 2.62(1.62)  [2.13, 3.11] | 2.60(1.75)  [2.13, 3.08] | 2.55(1.76)  [2.07, 3.02] | 2.89(1.79)  [2.42, 3.36] | 2.84(1.62)  [2.37, 3.03] | 2.70(2.04)  [2.21, 3.19] | 3.33(1.96)  [2.85, 3.81] | 2.58(1.70)  [2.09, 3.08] | 2.48(1.54)  [1.98, 2.98] | 2.69(1.82)  [2.20, 3.17] | 2.79(1.68)  [2.32, 3.27] |
| Year | 1956(21)  [1949, 1962] | 1956(21)  [1949, 1962] | 1961(24)  [1955, 1967] | 1955(24)  [1949, 1961] | 1968(27)  [1962, 1974] | 1961(25)  [1955, 1968] | 1969(26)  [1963, 1976] | 1962(24)  [1955, 1969] | 1967(24)  [1960, 1973] | 1963(23)  [1957, 1970] | 1955(13)  [1949, 1961] |
| Ease of Processing | 4.62(1.22)[4.27, 4.97] | 5.31(1.07)  [5.00, 5.60] | 4.15(1.36)  [3.77, 4.52] | 5.00(1.43)  [4.61, 5.39] | 4.98(1.32)  [4.62, 5.33] | 4.37(1.21)  [4.02, 4.70] | 4.64(1.40)  [4.24, 5.03] | 4.40(1.28)  [4.02, 4.77] | 4.17(1.26)  [3.81, 4.54] | 4.36(1.44)  [3.95, 4.76] | 5.01(1.26)  [4.66, 5.35] |

***Note***. PA=positive affect; NA=negative affect; RWA=right-wing authoritarianism; SDO=social dominance orientation; SA=self-authored.

**Supplemental Analyses, Study 2**

The text for the speeches used in the Pilot Studies and Study 2, as well as links to the original transcript of each speech are presented in Appendix A. Correlations among all measures can be found in Table S6.

**Preliminary Analyses**

As an initial pass through the data, we examined the unique effect of each passage. Figure S4 shows the means for meaning in life (top panel), arranged in descending order as a function of meaning in life (along with bootstrapped 95% confidence intervals). The bottom panel of the figures shows means for PA and NA for the corresponding messages. Means for meaning in life are adjusted for mood; and, those for mood are adjusted for meaning in life.

Controlling for meaning in life, authoritarian messages generally led to lower PA and higher NA, *F’s*(8, 1825)=23.04 for PA and 26.55, for NA, *d’s*= 0.64 and 0.68, respectively, *p’s*<.001. Speeches by all of the authoritarian leaders (and our authoritarian message) were lower than the egalitarian messages and the control passage on PA and higher on NA, *p*<.001, Bonferroni corrected.

For meaning in life, controlling for PA and NA, showed a significant effect of source of the message, *F*(8, 1824)=3.82, *d*=0.26, *p*<.001. As can be seen in Figure 1, the speech excerpt from Hitler led to the highest meaning in life. Post-hoc Bonferroni tests showed that the Hitler, Patton, and our authoritarian passages all led to significantly higher meaning in life than the control and our egalitarian passage. The egalitarian speeches, though lower than the authoritarian speeches, did not differ significantly from these.

Comparing the hedonic effect of Hitler’s speech to its effect on meaning in life is potentially illuminating. Note that participants in this condition reported significantly higher NA than PA, paired *t*(206)=2.79, *p*=.006 (PA and NA were unrelated, *r*=-.11, *p*=.11), *d*=0.22. Is it possible that the NA produced by the speech, itself, enhanced meaning in life? The relations between meaning in life and mood suggests that this is not the case. Among those who read the speech by Hitler, the correlation between NA and meaning in life was significant and negative, *r*=-.16, *p*=.025. In this condition, PA was positively related to meaning in life, *r*=.26, *p*<.001, as is typical (King & Hicks, 2020).

**Mood**

Because estimated means for meaning in life controlling for mood vs. raw estimates were higher in the authoritarian condition and lower in the egalitarian condition, we further probed the effects of condition and mood on meaning in life. Due to the large number of conditions, we determined to simply examine correlations between mood and meaning in life within each condition, rather than formally testing for moderation. Results are shown in Table S8. PA was positively associated with meaning in life in all conditions, and only the original authoritarian and egalitarian conditions significantly differed, *z* = 1.84, *p* = . 03. Negative affect was negatively related to meaning in life in all conditions. Although the relationship was non-significant in the original egalitarian condition, this relationship did not significantly differ from those obtained in any other condition.

**Other Variables**

Table S7 shows the results for mood, message evaluations, moral superiority, familiarity, relatedness, belongingness, religiosity and RWA. As predicted, the authoritarian conditions led to lower PA, higher NA, poorer evaluations, and greater moral superiority than the control and egalitarian conditions. There was one exception to this pattern: Egalitarian speeches did not differ from authoritarian passages on positive mood, though the original egalitarian passage did. Although both authoritarian conditions went in the same direction, authoritarian speeches led to lower positive affect, poorer evaluations, and greater moral superiority than the original passage. Speeches were experienced as less familiar than passaged we authored, likely due to the antiquated language contained in them. Authoritarian and egalitarian speeches did not differ on familiarity. The authoritarian, egalitarian, and control passages we authored did not differ from each other on familiarity, either. There were small effects of condition on religiosity (*p*=.044) and RWA (*p*=.032), such that the authoritarian conditions were slightly higher than egalitarian and control passages on these covariates.

**The Existential Vacuum**

We tested for moderation in support of the existential vacuum. We failed to find evidence of moderation by religious affiliation (*p*=.36) and conservatism, *p*’s<.60. Moderational results were marginal for right-wing authoritarianism. On the first step, Δ*R*^2^=.16, *p*<.001, main effects were significant for the authoritarian dummy, β= .16, *p*<.001, and RWA, β= .19, *p*<.001, and the egalitarian dummy did not contribute significantly, β= .03, *p*=.49. On the second step, the authoritarian condition X RWA interaction term was marginally significant, β= -.10, *p*=.07. The negative sign of the slope indicates that meaning was buffered at low levels of RWA in the authoritarian condition. The interaction term for the egalitarian condition was non-significant.

For religiosity, on the first step, Δ*R*^2^=.20, *p*<.001, main effects were significant for the authoritarian dummy, β= .15, *p*<.001, and religiosity, β= .28, *p*<.001, and the egalitarian dummy did not contribute significantly, β= .03, *p*=.57. On the second step, Δ*R*^2^=.01, *p*=.09, the authoritarian condition X religiosity interaction term was significant, β= -.11, *p*=.035. Again, the negative slope of the interaction term indicates that meaning in life was buffered by exposure to authoritarian values at low levels of religiosity.

**Message Evaluations**

We tested whether the increase in meaning in life after the manipulation led participants to evaluate the passages more positively. To simplify analyses, we collapsed the speeches and the original passages to create just 2 dummies, contrasted with the control group. In addition, because analyses showed no effects for the egalitarian condition, we focused on the authoritarian dummy. We regressed the message evaluation composite on mood Δ*R*^2^ = .34, *p*<.001, main effects (Δ*R*^2^ = .12, *p*<.001) for meaning in life, β=-.06, *p*=.009, the authoritarian dummy, β=-.36, *p*<.001 and finally the interaction of the authoritarian dummy with meaning in life (Δ*R*^2^=.002 *p*=.02), β=.054, *p*=.02. Meaning in life was more strongly related to positive evaluations in the authoritarian condition (*n*=811), β=.18, *p*<.001, than in the other conditions (*n*=1021), β=.10, *p*=.002, *z*=2.60, *p*<.001. These results suggest that those who experienced a meaning in life boost provided more positive evaluations of expressions of authoritarian values.

**Belongingness**

Participants completed 8 items measuring relatedness need satisfaction from the Basic Need Satisfaction Scale (Gagne, 2003; sample item: “People in my life care about me”), *M*(*SD*)=5.03(1.03), α=.82; and, the 6-item inclusion subscale of the General Belongingness Scale (Malone, Pillow, Osman, 2012; sample item: “I have close bonds with family and friends”), *M*(*SD*)=5.21(1.27), α=.94. These measures were strongly related, *r*=.79, *p*<.001 so they were standardized and aggregated into a belonging composite. Controlling for mood, the authoritarian condition led to significantly higher belongingness (the standardized composite) than the control condition, *M*=0.05 vs. -0.20, *F*(1, 1005)=10.43, *p*=.001, η^2^ = .011.

We tested for moderation of condition effects on meaning by belongingness, comparing only the authoritarian (dummy coded 1) to other conditions (coded 0). On the first step, Δ*R*^2^=.09, *p*<.001, effects for PA, β= .27, *p*<.001, and NA, β= -.11, *p*<.001, were significant. For the second step, Δ*R*^2^=.23, *p*<.001, main effects were significant for the authoritarian dummy, β= .06, *p*=.003, and the relatedness composite, β= .48, *p*<.001. On the final step, Δ*R*^2^=.002, *p*=.014, main effects were qualified by an authoritarian condition X relatedness interaction β=.06, *p*=.014. Generated regression lines are shown in Figure S5. As indicated by the Figure, belongingness was more strongly related to meaning in life in the authoritarian condition, β= .53, *p*<.001, than in the other conditions, β= .45, *p*<.001.

To test whether changes in belongingness might explain the effect of condition on meaning in life, we tested for mediation using PROCESS Macro for SPSS (Model 4, Hayes, 2016). In this model, we entered PA and NA as control variables and belongingness as a mediator of the effect of condition on meaning in life. For comparison purposes, in a model without belongingness, the direct effect of the manipulation on meaning in life, *b*=0.47(0.11), *p*<.001, 95% CI =[0.25, 0.70]. Results showed partial mediation. Controlling for belongingness, *b*=0.79(0.04), *p*<.001, 95% CI=[0.71, 0.87], the direct effect of exposure to the authoritarian message was weakened but remained significant, *b*=0.29(0.10), *p*=.003, 95% CI=[0.10, 0.48]. The indirect effect of belongingness was significant, *b*=0.19(0.06) 95% CI=[.08, .31]. Thus, the effect of condition is partially explained by its effect on feelings of relatedness to others.

***Table S6. Correlations Among Measures, Study 2***

|  | MIL | PA | NA | Eval | MS | Fam | RNS | Blong | Rel | Con |
| --- | --- | --- | --- | --- | --- | --- | --- | --- | --- | --- |
| RWA | .25** | .24** | -.03 | .13** | .09** | .16** | .02 | .11** | .59** | .65** |
| Meaning in Life |  | .28** | -.15** | .12** | .04 | .08** | . 45** | .55** | .34** | .22** |
| Positive Affect |  |  | -.13** | .52** | -.01 | .36** | .15** | .23** | .20** | .12** |
| Negative Affect |  |  |  | -.33** | .30** | .05 | -.12** | -.12** | .00 | -.06 |
| Evaluations |  |  |  |  | -.37** | .31** | .11** | .14** | .10** | .06* |
| Moral Superiority |  |  |  |  |  | .09** | -.09** | -.01 | .07* | .01 |
| Familiarity |  |  |  |  |  |  | -.04 | .02 | .13** | .08* |
| Relatedness |  |  |  |  |  |  |  | .79** | .14** | .06 |
| Belongingness |  |  |  |  |  |  |  |  | .20** | .12** |
| Religiosity |  |  |  |  |  |  |  |  |  | .40** |

***Note.*** **p*<.01; ***p*<.001. Pairwise *N*’s ranged from 1815-1820. MIL=meaning in life. PA= positive affect. NA= negative affect. Eval=evaluations composite. MS=moral superiority. Fam=familiarity. RNS=relatedness need satisfaction. Blong=belongingness. Rel=intrinsic religiosity. Con=conservatism.

***Table S7. Effect of Condition on Covariates, Study 2***

|  | **Control** | **Egalitarian SA** | **Egalitarian Speeches** | **Authoritarian**  **SA** | **Authoritarian Speeches** | **Effect of Condition** |
| --- | --- | --- | --- | --- | --- | --- |
| *n*’s | 212 | 213 | 640 | 209 | 630 |  |
| PA | 3.45(1.70)_a_ | 3.89(1.70)_a_ | 2.87(1.67)_b_ | 2.82(1.80)_b_ | 2.35(1.70)_c_ | *F*(4,1828)=39.11,* *d*=0.59 |
| NA | 1.70(1.05)_a_ | 1.66(1.03)_a_ | 1.85(1.14)_a_ | 2.40(1.51)_b_ | 2.63(1.53)_b_ | *F*(4,1828)=43.28,* *d*=0.63 |
| ME | 5.24(1.03)_a_ | 5.38(1.27)_a_ | 4.76(1.30)_b_ | 3.92(1.55)_c_ | 3.14(1.60)_d_ | *F*(4,1828)=176.66,* *d=*1.25 |
| MS | 2.58(1.55)_a_ | 2.56(1.63)_a_ | 2.67(1.57)_a_ | 3.21(1.68)_b_ | 3.87(1.86)_c_ | *F*(4,1828)=51.86,* *d*=0.67 |
| Fam | 3.20(1.79)_a_ | 3.15(1.93)_a_ | 2.39(1.59)_b_ | 3.09(1.86)_a_ | 2.20(1.59)_b_ | *F*(4,1828)=27.18,* *d*=0.51 |
| RNS | 4.82(0.98)_a_ | 4.99(1.04)_ab_ | 4.97(1.07)_ab_ | 5.14(0.98)_b_ | 5.12(1.02)_b_ | *F*(4,1828)=4.12*, *d*=0.20 |
| BL | 4.97(1.22)_a_ | 5.07(1.25)_ab_ | 5.15(1.27)_ab_ | 5.42(1.14)_b_ | 5.33(1.32)_b_ | *F*(4,1828)=5.27,* *d*=0.21 |
| REL | 3.11(2.10)_a_ | 2.95(2.04)_a_ | 3.28(2.05)_a_ | 3.40(2.03)_a_ | 3.43(2.04)_a_ | *F*(4,1828)=2.46, *d*=0.14 |
| RWA | 2.99(1.36)_a_ | 2.74(1.24)_a_ | 2.95(1.28)_a_ | 3.08(1.28)_a_ | 3.05(1.25)_a_ | *F*(4,1828)=2.65, *d*=0.16 |

***Note.*** **p*<.001. Means with differing subscripts significantly differed, *p*<.007. PA= positive affect. NA= negative affect. Eval=evaluations composite. MS=moral superiority. Fam=familiarity. RNS=relatedness need satisfaction. Blong=belongingness. Rel=intrinsic religiosity. Results for relatedness, belongingness, religiosity, and right-wing authoritarianism are shown controlling for mood.

**Table S8. Correlations between Mood and Meaning in Life within each Condition, Study 2**

|  | Authoritarian  Speeches | Original  Authoritarian | Control | Original Egalitarian | Egalitarian  Speeches |
| --- | --- | --- | --- | --- | --- |
| PA | .26*** | .37*** | .32** | .22** | .30** |
| NA | -.18*** | -.20** | -.21** | -.09 | -.11* |

***Note.*** *N*’s ranged from 210 to 612. ** *p* <.001; * *p* < .005. PA = positive affect; NA = negative affect

***Figure S4. Condition Effects on Meaning in life (controlling for mood; top panel) and Mood (controlling for meaning in life; bottom panel), Study 2***

***Note.*** Cell *n*’s range from 209 to 214. Error bars are bootstrapped 95% Confidence Intervals with 1000 resamplings.

**Figure S5. Belongingness Moderates the Effect of Condition on Meaning, Study 2**

***Appendix A***

**Adolf Hitler**

<https://carolynyeager.net/why-we-are-antisemites-text-adolf-hitlers-1920-speech-hofbräuhaus>

"Our enemy preaches every day with thousands of tongues that all nations on Earth are equal, that international solidarity should bind all the peoples, that no people can lay a claim to a special status etc., and, above all, that no nation has a reason to be proud of anything that is called or is national.

Common good before own good, a struggle against all parasites and especially against easy and unearned income. And in this fight we can rely on no one but our own people. Those who are not willing to fight for the blessings of that State, die."

**Kim Jong Un**

<https://www.ncnk.org/node/1427>

"All people should never tolerate all shades of different ideas and double standards of discipline that run counter to our ideology, but strengthen the single-hearted unity of the whole country in every way possible."

**George Patton**

<https://greatspeeches.wordpress.com/2008/09/30/pattons-speech-to-the-third-army-june-5th-1944/>

"We love a winner. We will not tolerate a loser. We despise coward. We play to win all of the time. I wouldn’t give a hoot in hell for a man who lost and laughed**.**That’s why we have never lost nor will ever lose a war; for the very idea of losing is hateful to us.

We don’t want yellow cowards in this Navy. They should be killed off like rats. If not, they will go home after this war and breed more cowards. The brave men will breed more brave men. Kill off the Goddamned cowards and we will have a nation of brave men."

**Dietrich Bonhoeffer**

<https://www.patheos.com/blogs/robertricciardelli/faith/dietrich-bonhoeffer-on-the-fuhrer-principle-by-bruce-norquist/>

"If a leader understands his function in any other way than as it is rooted in fact, if he does not continually tell his followers quite clearly of the limited nature of his lack and of their own responsibility, if he allows himself to surrender to the wishes of his followers, who would always make him their idol – then the image of the leader will pass over into the image of the misleader, and he will be acting in a criminal way not only towards those he leads, but also towards himself, the true leader must always be able to remind his followers that he is human and imperfect.  It is just this that is his responsibility and his real object.

He must lead his following away from the authority of his person to the recognitions of the real authority of orders and offices. He must radically refuse to become the appeal, the idol, i.e. the ultimate authority of those whom he leads."

**Mikhail Gorbachev**

<https://astro.temple.edu/~rimmerma/gorbachev_speech_to_UN.htm>

"Freedom of choice is a universal principle to which there should be no exceptions. This objective fact presupposes respect for other people’s views and stands, tolerance, a preparedness to see phenomena that are different as not necessarily bad or hostile, and an ability to learn to live side by side while remaining different and not agreeing with one another on every issue.

We are not giving up our convictions, philosophy, or traditions. Neither are we calling on anyone else to give up theirs. Yet we are not going to shut ourselves up within the range of our values. In the course of such sharing, each should prove the advantages of his own system, his own way of life and values by real deeds. That is, indeed, an honest struggle of ideology, but it must not be carried over into mutual relations between states."

**Eleanor Roosevelt**

<https://www.americanrhetoric.com/speeches/eleanorroosevelt.htm>

"Freedom, human rights have come to have a definite meaning to the people of the world which we must not allow any nation to so change that they are made synonymous with suppression and dictatorship. We in our country have come to realize it means freedom to choose one’s job, to work or not to work as one desires. We have come to realize, however, that people have a right to demand that their government will not allow them to starve because as individuals they cannot find work.

The basic problem confronting the world today is the preservation of human freedom for the individual and consequently for the society of which they is a part. The development of the ideal of freedom and its translation into the everyday life … is the fruit of a long tradition of vigorous thinking and courageous action. No one race and on one people can claim to have done all the work to achieve greater dignity for human beings and great freedom to develop human personality. In each generation and in each country there must be a continuation of the struggle and new steps forward must be taken since this is preeminently a field in which to stand still its to retreat. I think it is possible for us to maintain freedom and to do so peacefully and without recourse to force."

**Overview of Supplemental Analyses, Studies 3-5**

For readers interested in viewing raw data, distribution density plots for each condition on MIL in Studies 3-5 are shown in Figures S5, S6, and S7, respectively. All data may accessed online: <https://osf.io/me34h/?view_only=ed450f2a71e345b59dd6418f5c2821ca>.

For Studies 3-5, after testing our prediction, we tested whether conditions interacted with demographics. For regression equations, all continuous variables were mean centered or standardized. Main effects were entered on the first step and interaction terms on the second step of hierarchical regression equations. For all studies, conditions were represented by two dummy codes: egalitarian=1, all others=0; and authoritarian=1, all others=0. For categorical demographics, ANOVAs were used. Education was coded BA or greater=1; less than BA=0. Race/ethnicity was coded 1=White/0=non-White. Gender was coded 1=women; 0=men. Given the large *N*’s in most of the studies, as well as the exploratory nature of the moderational analyses, we urge caution in the interpretation of these results.

**Study 3 Supplemental Analyses**

Correlations among all measures are shown in Table S9. Condition effects on mood without controlling for meaning in life are shown in the top panel of Table S12.

**Demographics**. With regard to demographics, all analyses testing for moderation controlled for mood. First, there was a significant main effect for gender, *F*(1, 1470) = 7.63, *p* = .006, and condition, *F*(2, 1470) = 19.78, *p* < .001, η^2^ = .005 and but no evidence of moderation, *F*(2, 1470) = 2.25, *p* = .105, η^2^ = .003. For ethnicity, there was no significant main effect for ethnicity and no interaction of ethnicity X condition, *p*’s > .17, η^2’s^ < .002. For education, there were significant main effects, for education *F*(1, 1479) = 8.24, *p* = .004, η^2^ = .006 and condition, *F*(2, 1479) = 19.86, *p* < .001, η^2^ = .03, but no interaction, *F*(2, 1479) = 0.05, *p* = .95, η^2^ = .00.

For income, there were significant main effects of income, β = .13, *p* < .001 and authoritarian condition, β = .18, *p* < .001 (the egalitarian dummy was non-significant, β = .03, *p* = .23), and no evidence for moderation, *p*’s > .58. Age also failed to show evidence for moderation: Main effects for age, β = .17, *p* < .001, authoritarian condition, β = .17, *p* < .001, and egalitarian condition, β = .03, *p* = .229, and no significant interactions, *p*’s > .22.

***Table S9.* *Correlations among measures, Study 3***

|  | PA | NA | Message Evaluations | Moral Superiority |
| --- | --- | --- | --- | --- |
| MIL | .28** | -.16** | .11** | .07* |
| PA |  | -.10** | .55** | .05 |
| NA |  |  | -.37** | .36** |
| ME |  |  |  | -.26** |

***Note.*** *N*=1565. * *p*=.006, ***p*<.001. MIL = meaning in life; PA = positive affect; NA = negative affect; ME = message evaluations

***Figure S6. Distribution Density Plot for Meaning in Life by Condition, Study 3***


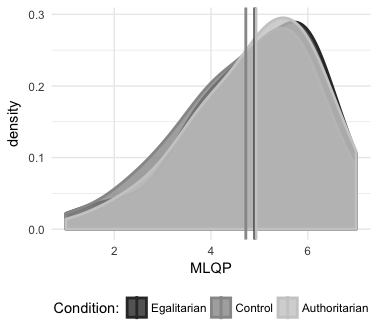


**Study 4 Supplemental Analyses**

Correlations among measures are shown in Table S10**.** Condition effects on mood without controlling for meaning in life are shown in the middle panel of Table S12.

**Demographics.** All analyses controlled for the effects of mood. For gender, there was no significant main effect or interaction, *p*’s > .14, η^2’s^ = .001; the main effect for condition remained significant, *F*(2, 1480) = 29.47, *p <* .001, η^2^ = .04, failing to replicate Study 3. For ethnicity, there was no significant main effect of ethnicity, a significant main effect of condition, *F*(2, 1488) = 30.57, *p <* .001, η^2^ = .03, and no significant interaction, *p* >.08, η^2’s^ < .003. For education, there was a significant main effect, *F*(1, 1492) = 15.78, *p* < .001, η^2^ = .011 and a significant main effect for condition, *F*(2, 1492) = 32.18, *p <* .001, η^2^ =.04; the education X condition interaction was not significant, *p* = .088, η^2^ = .003.

For income, there was a significant main effect, β = .12, *p <* .001, and a significant main effect for the egalitarian dummy, β = -.09, *p =* .002, and the authoritarian dummy, β = .15, *p <* .001, but no significant interaction; for income X authoritarian dummy, β =.01, *p = .*75*,* and for income X egalitarian dummy, β = .00, *p* = .98. Age did not show evidence of moderation: For the main effects of age, β = .11, *p* < .001, egalitarian condition, β = -.08, *p* = .006, authoritarian condition, β = .15, *p* < .001; and, the interaction of age X authoritarian condition, β = .06, *p* = .06, and age X egalitarian condition, β = .04, *p* = .25.

*Table S10. Correlations among measures, Study 4*

|  | PA | NA | Message Evaluations | Moral Superiority |
| --- | --- | --- | --- | --- |
| MIL | .25** | -.16** | .10** | .04 |
| PA |  | -.12** | .56** | .06* |
| NA |  |  | -.42** | .38** |
| Message Evaluations |  |  |  | -.25** |

*Note. N*=1505. **p*=.027, ***p*<.001. MIL = meaning in life; PA = positive affect; NA = negative affect.

***Figure S7. Distribution Density Plot for Meaning in Life by Condition, Study 4***


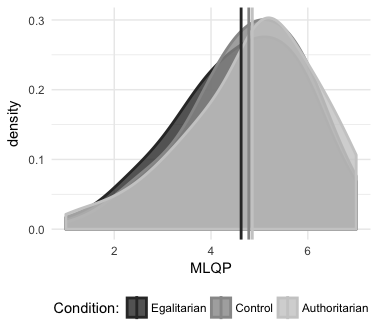


**Study 5 Supplemental Analyses**

Correlations among measures are shown in Table S11. Condition effects on mood without controlling for meaning in life are shown in the bottom panel of Table S12.

**Were Canadians less conservative than the American samples?** Canadians rated themselves as significantly less conservative *M*(*SD*)=3.01(1.56) than the Americans in the other studies, *M*(*SD*)=3.59(1.69), *t*(4659)=4.07, *p*<.001, *d*=0.12.

**Condition Effects on RWA.** To test for main effects of condition on RWA, we submitted RWA to an ANCOVA, controlling for PA and NA. Although RWA was slightly higher in the authoritarian condition, *M(SD)* = 2.75(1.05), than the egalitarian, *M(SD)* = 2.45(0.98), and control, *M(SD)* = 2.52(0.09), conditions, these differences were not significant, *F*(2,143) = 1.79, *p* = .17, η^2^ = .024.

It might be tempting to assume that those low in RWA were energized by negative feelings engendered by the authoritarian message. However, neither negative affect (generally) nor anger (specifically) interacted with RWA and condition to predict MIL in a manner that fits this interpretation. In any case, these results should be viewed with caution as the Preliminary Study had the power to detect the effect but used a limited measure of RWA; Study 5 lacked power but used a superior measure of RWA.

**Demographics.** For demographics, again all analyses controlled for mood. There was no evidence for moderation by gender, ethnicity, education, income, and age. For intrinsic religiosity, on the first step, Δ*R*^2^ = .24, *p* < .001, religiosity, β = .24, *p* = .002, the authoritarian dummy, β = .32, *p* < .001, and PA, β = .30, *p* < .001 contributed significantly (the effects of the egalitarian dummy, β = .11, *p* = .21, and NA, β = -.10, *p* = .19 did not reach significance). On the second step, Δ*R*^2^ = .02, *p* = .13, we found a marginal intrinsic religiosity X authoritarian condition interaction, β = -.23 *p* = .047 (the religiosity X egalitarian interaction was non-significant, β = -.09, *p* = .44). Generated regression lines for those +/- 1 *SD* from the mean on intrinsic religiosity at both levels of the authoritarian dummy are shown in Figure S9. As can be seen in the Figure, religiosity was less strongly related to meaning in the authoritarian condition, β = .09, *p* = .51, than in the other conditions, β = .35, *p* < .001, because those low on religiosity were particularly strongly affected by exposure to authoritarian values.

**Moderation by Right-wing Authoritarianism.** We next tested whether right-wing authoritarianism moderated the effect of condition on meaning in life. In this sample, as in past research, right-wing authoritarianism was correlated with meaning in life, *r*(148)=.29, *p<*.001. Meaning in life was regressed on mood, mean-centered right-wing authoritarianism, dummy codes for condition and the interaction of right-wing authoritarianism and condition dummies. On the first step, Δ*R*^2^ = .23, *p* < .001, all variables except NA and the egalitarian dummy contributed significantly: PA (β = .30, *p* < .001), NA (β = -.11, *p* = .17), authoritarian dummy (β = .11, *p* = .001), egalitarian dummy (β = .10, *p* = .27), and right-wing authoritarianism (β = .22, *p* = .005). On the second step, Δ*R*^2^ = .04, *p* = .021, main effects were qualified by a significant right-wing authoritarianism X authoritarian condition interaction, β = -.27, *p* = .02 (the right-wing authoritarianism X egalitarian condition did not contribute significantly, β = .02, *p* = .87). The generated means for participants +/- 1 *SD* from the mean on right-wing authoritarianism are shown in Figure S10. As can be seen, condition effects were strongest among those *low* in right-wing authoritarianism. Among those high in right-wing authoritarianism, meaning in life was high regardless of condition.

**Table S11. *Correlations among measures, Study 5***

|  | PA | NA | Passage Evaluations | Moral Superiority | RWA | Intrinsic Religiosity |
| --- | --- | --- | --- | --- | --- | --- |
| MIL | .32** | -.05 | .10 | .18* | .29** | .29** |
| PA |  | -.10 | .58** | -.02 | .07* | .06 |
| NA |  |  | -.42** | .33** | .12 | .09 |
| Passage Evaluations |  |  |  | -.36** | .20* | .14 |
| Moral Superiority |  |  |  |  | .18* | .01 |
| RWA |  |  |  |  |  | .56** |

***Note.*** *N*=148. **p*<.05, ***p*<.001. MIL = meaning in life; PA = positive affect; NA = negative affect

***Figure S8. Distribution Density Plot for Meaning in Life by Condition, Study 5***


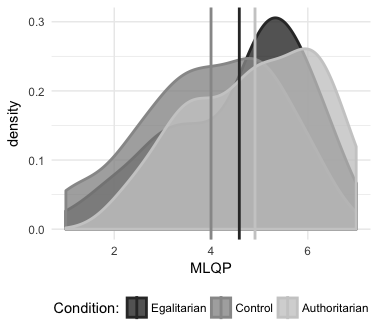


***Figure S9. Intrinsic Religiosity Moderates the Effect of Condition on Meaning, Study 5***

***Figure S10. Meaning in life as a function of authoritarian condition and right-wing authoritarianism, Study 5***

***Table S12. Effects of Condition on Mood, Studies 3, 4, and 5***

| **Study** |  | **Control** | **Egalitarian** | **Authoritarian** | **Effect of Condition** |
| --- | --- | --- | --- | --- | --- |
| 3 | *n*’s | 519 | 527 | 527 |  |
|  | PA | 3.55(1.68)_a_ | 4.00(1.68)_b_ | 2.98(1.77)_c_ | *F*(2,1564)=45.54,** *d*=0.51 |
|  | NA | 1.87(1.23)_a_ | 1.80(1.21)_a_ | 2.66(1.60)_b_ | *F*(2,1564)=64.70,** *d*=0.59 |
| 4 | *n*’s | 505 | 506 | 501 |  |
|  | PA | 3.61(1.67)_a_ | 3.94(1.60)_b_ | 2.74(1.62)_c_ | *F*(2,1509)=72.88,** *d*=0.63 |
|  | NA | 1.78(1.18)_a_ | 1.81(1.16)_a_ | 2.71(1.62)_b_ | *F*(2,1509)=78.03,** *d*=0.63 |
| 5 | *n*’s | 46 | 52 | 50 |  |
|  | PA | 3.05(1.50)_a_ | 4.02(1.30)_b_ | 2.89(1.47)_c_ | *F*(2,145)=9.34,** *d*=0.70 |
|  | NA | 2.18(1.30)_a_ | 1.93(1.36)_a_ | 2.73(1.37)_b_ | *F*(2,145)=4.71,† *d*=0.51 |

***Note.*** PA=Positive Affect, NA=Negative Affect. †*p*=.01; **p*=.004; ***p*<.001. Means in the same row with differing subscripts are significantly different, *p*<.05, Bonferroni corrected.

**Moderation by Conservatism, Studies 3-5**

In all studies, we tested whether liberals were more likely to find existential appeal in exposure to authoritarian values, given they lack dispositional sources of meaning (i.e., conservatism is dispositionally positively related to meaning in life, Newman et al., 2019).

To test this possibility, we pooled data from Studies 3-5. Collapsed across cells, as expected, meaning in life was positively related to conservatism, *r(*3133*)*=.20, *p*<.001. We mean-centered conservatism, and computed interaction terms using dummy variables representing the authoritarian and egalitarian conditions. Then, we regressed meaning in life on positive and negative affect and the main effects for conservatism and condition on the first step and interaction terms on the second step.

On Step 1, Δ*R*^2^=.14, *p*<.001, the main effects of authoritarian condition, β=.18, *p*<.001, and conservatism, β=.17, *p*<.001 (for PA, β=.27, *p*<.001, and NA, β=-.17, *p*<.001) were significant. On the second step, Δ*R*^2^=.01, *p*<.001, main effects were qualified by a significant conservatism X authoritarian condition interaction, β=-.10, *p*<.001. Generated regression lines for those +/- 1 *SD* from the mean on conservatism in the authoritarian vs. other conditions are shown in Figure S11. The Figure shows that the effect of exposure to authoritarian values on meaning in life was particularly strong for those *low* on conservatism. Conservatism was less strongly related to meaning in life in the authoritarian condition, β=.09, *p*=.003, than in the other conditions, β=.23, *p*<.001, *z*=-3.80, *p*<.001, because meaning in life was high across all levels of conservatism in this condition.

With regard to the idea of an existential vacuum, results supported the idea that exposure to authoritarian values led to higher meaning in life particularly for those low on conservatism. It might be assumed that meaning in life would be enhanced by exposure to messages consistent with one’s preexisting worldview. In contrast, we found that it was individuals who were low on conservatism who experienced the largest boost in existential meaning in response to messages conveying authoritarian values. Conservatives generally already agree with these messages and endorse high levels of meaning in life, representing a ceiling effect. In contrast, it was those who do not already endorse such beliefs, who showed the largest boost in meaning in life. It is likely that this expression of authoritarian values represented an expression of extreme ideological thinking (involving descriptive and prescriptive dogma, as well as in-group favoritism and out-group animus, Zimgrod, 2021).It may be that any expression of extreme ideology, regardless of content, promotes meaning in life, a possibility that should be probed by future research.

***Figure S11.* Moderation of Condition Effects on Meaning in Life by Conservatism, Merged Data from Studies 3 through 5**

**Testing Moderation of Condition Effects on Meaning in Life by Mood and Covariates, Preliminary Study and Studies 3-5**

Because in Studies 3-5 and the Preliminary Study, condition affected all covariates, condition and these continuous variables were not independent. We first standardized all covariates within condition (removing mean differences). Then, we probed for interactions.

Table S13 shows the within cell correlations for all covariates and the results for General Linear Models testing for Condition X Covariate interactions for Studies 3-5 and the preliminary Study. Clearly, moderational results were weak and inconsistent. In the preliminary Study (authoritarian dummy only) and Study 3 (both condition dummies), negative affect interacted with condition to predict MIL. As shown in Figure S12, conditions wiped out the negative association between negative affect and MIL that occurred in the control condition. Those that were high on NA in the authoritarian and egalitarian conditions were relatively high on MIL compared to the control condition. In addition, in the Preliminary Study and Study 3, condition interacted with message evaluations. Slopes predicting MIL are shown in Figure S13. As shown in the Figure, these results were inconsistent across studies. For the preliminary Study, those in the egalitarian condition showed lower MIL when they evaluated the message more positively. In contrast, in Study 3, those in the egalitarian condition showed higher MIL when they evaluated the message favorably. Finally, in Study 3, PA interacted with condition. Figure S14 shows the slopes predicting MIL. Positive affect was more strongly related to MIL in the control condition than the other conditions. Those in the authoritarian and egalitarian conditions were relatively high on MIL at low levels of PA.

***Table S13. Correlations Between Covariates and Meaning in Life within Conditions, Preliminary Study and Studies 3-5.***

| **Study** |  | **Control** | **Egalitarian** | **Authoritarian** | **Condition X Covariate^1^** |
| --- | --- | --- | --- | --- | --- |
| Pilot | *n*’s | 535 | 544 | 532 |  |
|  | PA | .25** | .13* | .14** |  |
|  | NA | -.18** | -.09 | -.08 | *F*(2,1588)=3.38, *p*=.034, partial η^2^=.004 |
|  | ME | .17** | -.05 | .12 | *F*(2, 1588)=8.47, *p*<.001, partial η^2^=.011 |
|  | MS | .12 | -.03 | .08 |  |
| 3 | *n*’s | 519 | 527 | 527 |  |
|  | PA | .39** | .21** | .28** | *F*(2,1550)=3.12, *p*=.045, partial η^2^=.004 |
|  | NA | -.27** | -.13* | -.15** | *F*(2,1550)=7.35, *p*=.001, partial η^2^=.009 |
|  | ME | .20** | .01 | .21** | *F*(2,1550)=3.83, *p*=.022, partial η^2^=.005 |
|  | MS | .08 | .09 | .02 |  |
| 4 | *n*’s | 505 | 506 | 501 |  |
|  | PA | .32** | .23** | .29** |  |
|  | NA | -.24** | -.14** | -.19** |  |
|  | ME | .09 | .06 | .23** |  |
|  | MS | .03 | .10 | -.04 |  |
| 5 | *n*’s | 46 | 52 | 50 |  |
|  | PA | .30* | .36* | .38* |  |
|  | NA | -.20 | -.05 | -.04 |  |
|  | ME | .13 | .12 | .26 |  |
|  | MS | .15 | .14 | .11 |  |

***Note.*** PA=Positive Affect, NA=Negative Affect. ME=Message Evaluation. MS=Moral Superiority. **p<*.01; ***p*<.001. ^1^Only significant results are shown.

***Figure S12*. *Condition X Negative Affect Predicting Meaning in Life, Preliminary Study, and Study 3***

***Figure S13.*** ***Condition X Message Evaluations, Preliminary Study, and Study 3***

***Figure S14. Meaning in Life as a Function of PA and Condition, Study 3***

**Moderation of Condition Effects on Message Evaluations, Studies 3-5**

Following the same procedure reported in the text, we merged data from Studies 3-5 and tested for moderation of condition effects on message evaluations by mood. We mean-centered positive and negative affect and used these to compute interaction terms with dummy codes representing condition. On the first step, Δ*R*^2^=.47, *p*<.001, the main effect for PA, β=.49, *p*<.001, NA, β=-.24, *p*<.001, and authoritarian condition, β=-.26, *p*<.001 contributed significantly (there was no effect of egalitarian, β=.03, *p*=.24). On the second step, Δ*R*^2^=.02, *p*<.001, main effects were qualified by significant PA X authoritarian condition, β=.18, *p*<.001, and a marginal PA X egalitarian condition, β=.06, *p*=.04, interaction. Generated regression lines for those +/- 1 *SD* from the mean on PA in each condition are shown in Figure S15. The Figure shows that PA was more strongly related to evaluations in the authoritarian condition, β=.60, *p*<.001, than in the egalitarian condition, β=.52, *p*<.001, and control condition, β=.45, *p*<.001. Negative affect showed no evidence of moderation, all *p*’s>.41.

***Figure S15. Positive Affect X Condition Predicting Message Evaluations, Studies 3-5***

**Results for Moral Superiority, Studies 2-5**

One item, “I am morally better than the writer of this essay,” was used to gauge whether results were due to downward social comparison with the authoritarians. Results for condition effects on moral superiority in Studies 2-5 are shown in Tables S14 and S15. Participants reported being more morally superior to the authors of the authoritarian speeches and our authoritarian passage in all studies. Moral superiority was unrelated to meaning in life in all Studies (*p*’s > .05).

***Table S14. Moral Superiority, Study 2***

|  | **Control** | **Egalitarian Speeches** | **Original**  **Egalitarian** | **Authoritarian Speeches** | **Original**  **Authoritarian** | **Effect of Condition** |
| --- | --- | --- | --- | --- | --- | --- |
| *n*’s | 212 | 640 | 213 | 630 | 209 |  |
| MS | 2.58(1.55)_a_ | 2.67(1.57)_a_ | 2.56(1.63)_a_ | 3.87(1.86)_c_ | 3.21(1.68)_b_ | *F*(4,1828)=51.86,* *d*=0.67 |

***Note.*** **p*<.001. Means in the same row with differing subscripts significantly differed, *p*<.007, Bonferroni adjusted. MS=moral superiority.

***Table S15. Moral Superiority, Studies 3, 4, and 5***

| **Study** |  | **Control** | **Egalitarian** | **Authoritarian** | **Effect of Condition** |
| --- | --- | --- | --- | --- | --- |
| 3 | *n*’s | 519 | 527 | 527 |  |
|  | MS | 2.52(1.54)_a_ | 2.55(1.67)_a_ | 3.29(1.74)_b_ | *F*(2,1562)=36.70,** *d=*0.46 |
| 4 | *n*’s | 505 | 506 | 501 |  |
|  | MS | 2.49(1.57)_a_ | 2.61(1.66)_a_ | 3.18(1.67)_b_ | *F*(2,1502)=25.17*,** d*=0.35 |
| 5 | *n*’s | 46 | 52 | 50 |  |
|  | MS | 2.33(1.65)_a_ | 2.29(1.41)_a_ | 3.22(1.567)_b_ | *F*(2,145)=5.88**, d=*0.59 |

***Note.*** MS=Moral Superiority. †*p*=.01; **p*=.004; ***p*<.001. Means in the same row with differing subscripts are significantly different, *p*<.05, Bonferroni corrected.

**Probing the Effects of Condition on Mood and Meaning in Life in the Aggregate Data, Studies 3-5**

For comparison purposes we repeated the main analyses in this pooled data. For PA, controlling for meaning in life, the effect of condition was significant, *F*(2,3224)= 145.61, *p*<.001, *d*=0.59. The egalitarian condition was significantly higher on PA, *M*(*SD*)=3.98(1.62) than either than controls M(SD)=3.58(1.67) and authoritarian M(SD)=2.83(1.69). The control was higher than authoritarian, all *p’s*<.001, Bonferroni corrected. Similarly, controlling for meaning in life, the effect of condition on NA was significant, *F*(2,3223)=160.41, *p*<.001 , *d*=0.63. The authoritarian condition was higher in NA, *M*(*SD*)=2.70(1.4) than both controls *M*(*SD*)=1.84(1.21) and egalitarian *M*(*SD*)=1.81 (1.19), *p*<.001. Controls and egalitarian did not differ from each other. Finally, controlling for mood, the effect of condition on meaning in life was significant, *F*(2, 3222)=50.97, *p*<.001, *d*=0.36. Bonferroni corrected pairwise comparisons showed that the authoritarian condition, *M(SD*)=5.13(1.34) was significantly higher than the control condition, *M(SD*)=4.65(1.32), *p*<.001, and the egalitarian condition, *M(SD*)=4.59(1.35), *p*<.001, which did not differ from each other, *p*=.82.

**Further Probing the Potential Effect of Anger and Agreement, Pooled Data, Studies 3-5**

These studies show that exposure to authoritarian values leads to worse mood and enhanced meaning in life. An intuitively appealing explanation for these results is that feelings of reactance, revulsion, enemyship, or anger [52. 53, 54] in response to the authoritarian message energized people, enhancing meaning in life. We probed this explanation using the pooled data set and found no support for it. In no case did anger or negative affect (as shown above) relate to meaning in life with different sign or magnitude across conditions. Such an explanation would imply that meaning in life is not enhanced by the content of authoritarian messages but rather by high activation negative affect that occurs in reaction against the message. Next, we sought to probe this explanation.

First, focusing on the anger item in the NA composite, we found that anger was significantly higher in the authoritarian condition *M (SD)*  = 2.50( 1.79) compared to the egalitarian 1.61 (1.24) and control conditions 1.52 (1.65) Bonferroni corrected *p* <.001, *F*(2,3224) = 154.35, *p < .*001, *d* = 0.62. Regression analyses showed that neither the egalitarian nor authoritarian condition dummies interacted with anger to predict meaning in life (β’s = .07, *p* = .045 for the authoritarian dummy X anger and β = .05, *p* = .063 for the egalitarian dummy X anger interactions). Nevertheless, to rule out the idea that anger might boost meaning in life in the authoritarian condition, we calculated the correlation between anger and meaning in life in each condition separately. Anger was negatively correlated with meaning in all conditions: for the egalitarian condition, *r*(1085) = -.10, *p* = .002; for the control condition, *r*(1067) = -.17, *p* <.001; and for the authoritarianism condition, *r*(1075) = -.14 *p < .*001. Now, anger may be ambiguous in this context. People might be inspired by the authoritarian message and share in the hostile attitude of the author or they may be angered by the message content. We undertook additional analyses to address this potential explanation using agreement with the passage and anger. In no case did the results support this account of the boost in meaning life caused by the authoritarian message. Even in the context of low agreement (among those 1-*SD* below the mean on agreement with the authoritarian message), anger was not positively related to meaning in life *r*(240) = -.10, *p* = .14. See the Supplement (p. 62-64) for full results of these analyses.

Due to its intuitive appeal, we further probed the possibility that disagreement and anger could illuminate the effect of condition on meaning in life. This explanation entails interactions of agreement with condition (such that disagreement would predict higher meaning in life in the authoritarian condition), negative emotion (particularly anger) with condition (such that anger would be positively correlated with meaning in life in the authoritarian condition), or a three-way interaction, between the authoritarian condition, agreement, and negative affect. Using data pooled from Studies 2-5, we tested these possibilities.

For each study, we regressed meaning in life on the dummy variables for condition, the item, “I agreed with the writer’s beliefs in the essay,” and a composite of the two anger-related negative affect items (angry, frustrated), and all possible interactions. Because even with large samples, a 3-way interaction may be difficult to detect, we also conducted these analyses using aggregated data from Studies 2-5 (*N*=5,027*)*. Table S15 shows that no three-way interactions involving the authoritarian condition emerged in any study or the merged dataset. In the merged dataset, the Jeffrey-Zellner-Siow Bayes Factor (Rouder, Speckman, Sun, Morey, & Iverson, 2009) for the 3-way step indicates the null to be nearly 7 times more likely than the alternative of a three-way interaction.

One result in Table S16 might seem to support the idea that anger or disagreement might boost meaning in life in the authoritarian condition--the significant anger X authoritarian dummy interaction in Study 4 and the merged dataset. Was anger positively related to meaning in life in the authoritarian condition? It was not. Anger was negatively related to meaning in life, in the authoritarian condition, in Study 4 *r*(500)=-.20; and in the merged dataset *r*(1887)=-.16, *p*’s<.001. Across all studies, the relationship between anger and meaning in life in the authoritarian conditions ranged from *r*(501)=-.20 (Study 4) to *r*(50)=.07 (Study 5). In the other conditions, these correlations ranged from *r*(1011)=-.16 (Study 4) to *r*(527)=-.10 (Study 3). Feeling angry never predicted higher meaning in life.

***Table S16. Meaning as a Function of Condition, Anger, and Agreement, Studies 2-5***

|  | Step 1 Δ*R*^2^ | Auth β | Egal β | Anger β | Agree β | Step 2 Δ*R*^2^ | Anger X Agree β | Anger X Auth β | Agree X Auth β | Anger X Egal β | Agree X Egal β | Step 3 Δ*R*^2^ | Anger X Agree X Auth β | Anger X Agree X Egal β |
| --- | --- | --- | --- | --- | --- | --- | --- | --- | --- | --- | --- | --- | --- | --- |
| Study 2 | .10** | .20** | .13* | -.19** | .04 | .003 | -.14 | .05 | -.07 | .04 | -.10 | .001 | .13 | .04 |
| Study 3 | .11** | .15** | .06* | -.26** | .03 | .02** | -.07 | .08 | -.04 | .04 | -.15** | .001 | .02 | -.02 |
| Study 4 | .12** | .13** | -.07* | -.33** | -.11 | .01 | .13 | .12* | .03 | .08* | -.06 | .004* | -.13 | -.10* |
| Study 5 | .18** | .29* | .13 | -.21** | .06 | .05 | -.16 | .10 | .01 | .11 | -.11 | .001 | -.08 | -.01 |
| Merged | .10** | .10** | -.01 | -.15** | -.09** | .01** | -.06** | .08* | -.01 | .07* | -.08* | .00 | -.03 | -.05 |

***Note.*** Study 2 *N*=1829; Study 3 *N*=1554; Study 4 *N*=1497; Study 5 *N*=146; Merged *N*=5027. **p*<.018, ***p*<.001. Auth=authoritarian condition dummy (1=authoritarian, 0=other). Egal= egalitarian condition dummy (1=egalitarian, 0=other). Anger = composite of two negative affect items (frustrated and angry): Study 2 *M(SD*)=2.18(1.51), inter-item *r*=.71; Study 3 *M(SD*)=2.08(1.50), inter-item *r*=.78; Study 4 *M(SD*)=2.03(1.44), inter-item *r*=.74; Study 5 *M(SD*)=2.17(1.48), inter-item *r*=.76. In all models, we controlled for positive affect on the first step.
