# Supplementary material for: Exposure to authoritarian values leads to lower positive affect, higher negative affect, and higher meaning in life
Source: PLoS One. 2021 Sep 15;16(9):e0256759. doi: 10.1371/journal.pone.0256759 (PMC8443031; doi:10.1371/journal.pone.0256759)
Supplement: Attachment — Submitted filename: Response to Reviewers.docx [file pone.0256759.s003.docx]

Dear Dr. Holtgraves,

Thank you for again providing thoughtful and constructive comments on our manuscript, PONE-D-21-00255R1) "Exposure to Authoritarian Values Leads to Lower Positive Affect, Higher Negative Affect, and Higher Meaning in Life." We have completed revision in response to each of your comments, which we believe continue to improve the quality of our work. Below we detail each of these revisions:

1. First, for each study, please indicate when (month and year) the data was collected.

**Response**: These have been added for each Study.

2. Second, I’m a little confused about your response to reviewer 1’s question about testing the equality of slopes for your ANCOVAs (reviewer 4 had a somewhat related concern).  Note that you don’t actually say ANCOVA in the manuscript, but I’m assuming that’s what you’ve ran.  In your revision, please be more specific about the type of analyses you conducted (for all of your analyses). In response to this reviewer’s comment, you report multiple regression analyses in Table 5 designed to assess the possible moderation of condition on meaning in life by mood.  Although potentially interesting, this doesn’t really address the question of equality of slopes (it hints at it but doesn’t directly address it).  The point is that your main analyses testing for condition on mood controlling for meaning, and condition on meaning controlling for mood, require a test of the covariate by condition interaction to test for slope equality. This is especially important because you rely on means that are adjusted by the covariate(s), and this can be misleading if the slopes are unequal. So, please test and if the interactions are not significant, everything can be reported as is (but please note in your manuscript that this assumption was tested).  If any of the interactions are significant, then the reporting of the adjusted means becomes a little more problematic. There are various options when the interaction is significant, the most common being to test the IV at different levels of the covariate (e.g., the mean and one SD above and one SD below), similar to what you do in the analyses you report in figures 3 - 5.  Another option would be to report only the unadjusted means and corresponding tests. There may be other options as well.

**Response**: We ran moderated regression models to test for interactions of these variables with condition to address whether slopes for mood differed across conditions. *Z*-scores are provided testing for these differences. These were already reported for Study 2 on p. 23 of the Supplement. We have added additional information for Studies 3-5 on p. 30 of the main text. These results show that in no case did the slopes in the authoritarian vs. egalitarian conditions significantly differ. In Study 3 (only), NA was more strongly negatively related to meaning in life in the *control* condition than in the authoritarian condition, *z* = 2.21, *p* = .027. We do not view this result as particularly informative. Note as well that it occurred in a direction opposite of the notion that enhanced anger in response to the *authoritarian* condition boosted meaning in life. Considering results from all of the studies together, these data suggest that the relationship between mood and meaning in life did *not* vary across conditions. Additionally, throughout the manuscript we have explicitly stated each type of analysis conducted.

3. Third, the df in Table 4 seem off (e.g., for Study 5 the df for the test of the adjusted means cannot be correct).  Please verify all values in this (and all other) tables.

**Response**: Thank you for your close attention to our work and for catching this error. We have corrected the values in Table 4, and verified the other values are correct.

Thank you again for the continued opportunity for our work to be considered for publication.

Sincerely,

Jake Womick
